# Supplementary material for: ELMO1 Deficiency Reduces Neutrophil Chemotaxis in Murine Peritonitis
Source: Int J Mol Sci. 2023 Apr 30;24(9):8103. doi: 10.3390/ijms24098103 (PMC10179205; doi:10.3390/ijms24098103)
Supplement: Supplementary file 1 [file ijms-24-08103-s001.zip › ijms-2316658-supplementary.pdf]

Supplementary Figure S1

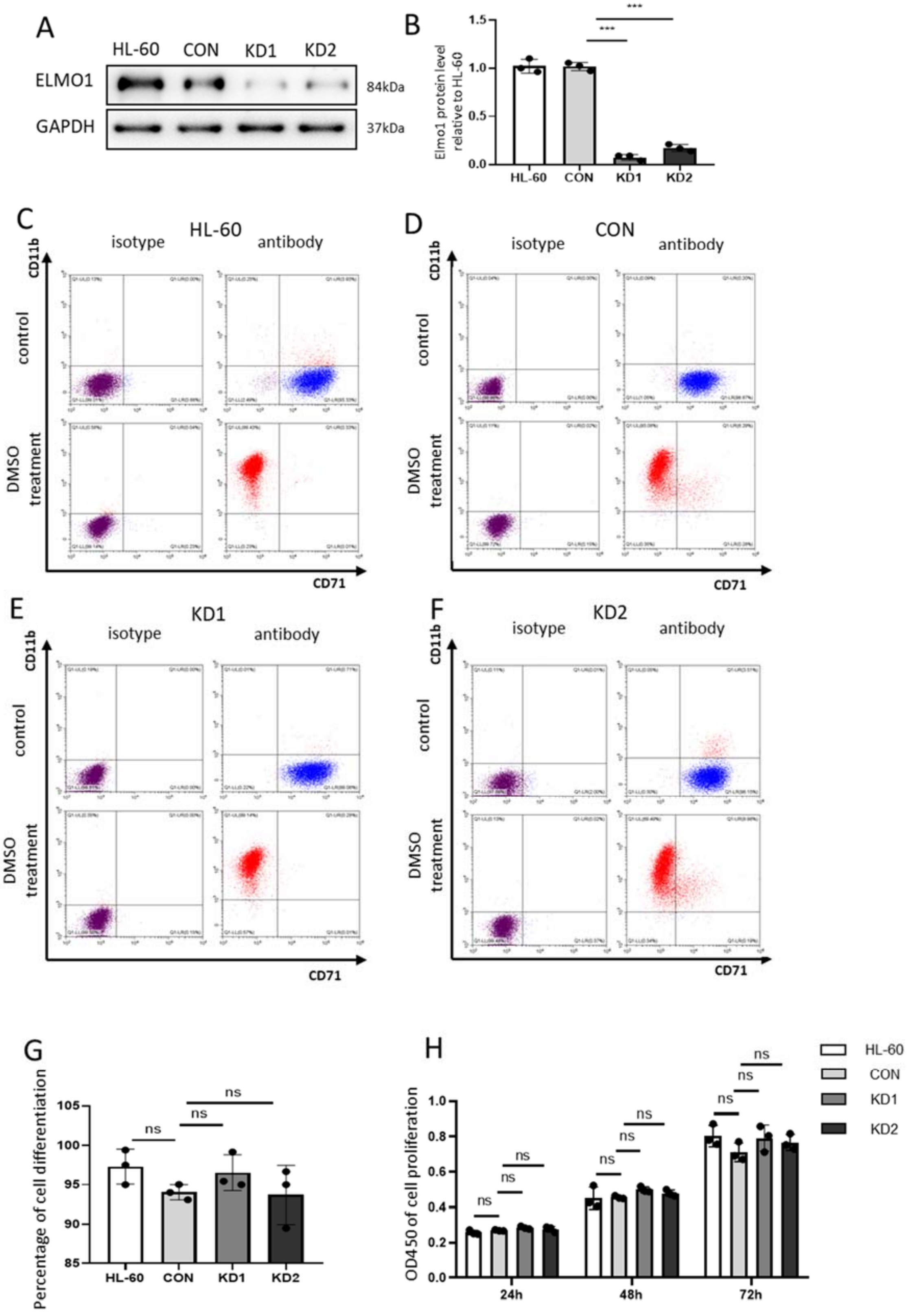

Figure S1. (A-B) ELMO1 protein in HL-60 cells were subjected to SDS-PAGE,

quantified and graphed. (C-F) HL-60 cells differentiation assay. DMSO-induced neutrophil differentiation were stained with CD11b and CD71 to detect the ratio by flow cytometry. One representative result is shown. (G) The percentage of CD11b and CD71 positive cells were quantified with FlowJo software and the data was indicated as means ± SEM (n = 3). (H) HL-60 cells proliferation assay. Cells were incubated at 37°C for indicated time and stained with CCK-8 for measuring absorbance at 450 nm. The data was indicated as means ± SEM (n = 6). Each group had 3 replicates. Statistical significance was assessed by *t*-test, KD groups versus CON group by one-way ANOVA. $^{*}P < 0.05$, $^{**}P < 0.01$, $^{***}P < 0.001$, *and* $^{ns}P > 0.05$.

Supplementary Figure S2

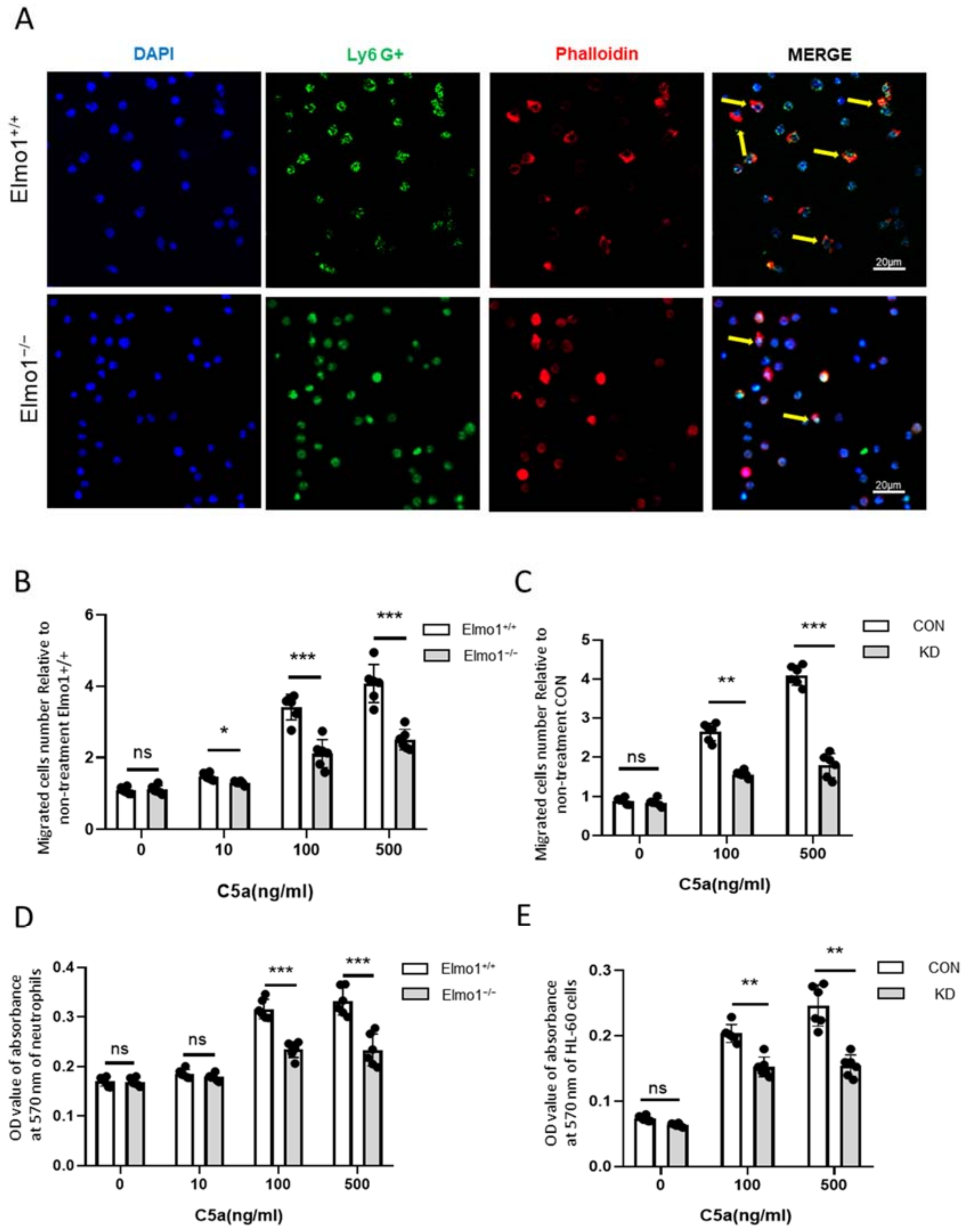

Figure S2. (A) Neutrophil F-actin polymerization assay. Confocal micrographs of fMLP-stimulation cells stained with Alexa Fluor 633 phalloidin, $Ly6G^{+}$ and DAPI. (B-C) Primary neutrophils and HL-60 cells migration assay (n = 6). Cells migrate under stimulation of C5a for 2 h. Migrated cell numbers in the bottom chamber were counted by flow cytometry. The data was indicated as means ± SEM. (D-E) Primary neutrophils and HL-60 cells adhesion assay. The C5a-stimulated cells

were incubated at 37℃ for 2 h, and stained with crystal violet followed by 2% SDS dissolving for measuring absorbance at 570 nm. The data was indicated as means ± SEM (n = 5). Each group had 3 replicates. Statistical significance was assessed by *t*-test. $^{*}P < 0.05$, $^{**}P < 0.01$, $^{***}P < 0.001$, *and* $^{ns}P > 0.05$.

Supplementary Figure S3

A

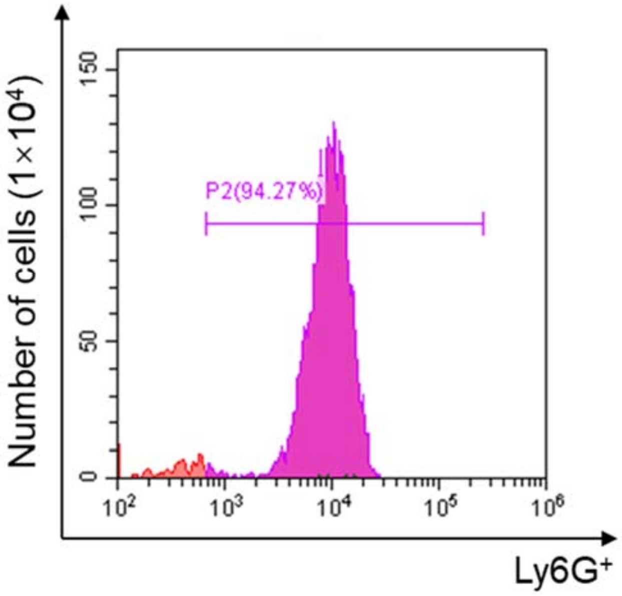

Figure S3. (A) Neutrophil purity assay. Cells from the middle layer of percoll™ were stained with $Ly6G^+$ to detect the positive ratio by flow cytometry. One representative result is shown.
